# Biphasic Flow-Volume Loop in a Patient With Idiopathic Unilateral Mainstem Bronchus Obstruction

**DOI:** 10.7759/cureus.21646

**Published:** 2022-01-26

**Authors:** Christopher K Hwe, Krista Dollar, David W Hsia

**Affiliations:** 1 Division of Respiratory and Critical Care Medicine, Harbor-UCLA Medical Center, Torrance, USA; 2 Department of Medicine, Harbor-UCLA Medical Center, Torrance, USA; 3 Division of Respiratory and Critical Care Medicine, The Lundquist Institute for Biomedical Innovation, Torrance, USA

**Keywords:** non-malignant, pulmonary function testing, spirometry, central airway obstruction, tracheal bronchus, airway stenosis

## Abstract

Unilateral mainstem obstruction is an uncommon cause of dyspnea in the clinic setting. However, it is identifiable on spirometry as the “two-compartment phenomenon,” in which the expiratory and/or inspiratory flow is decreased, followed by a further rapid decrease, resulting in a flattened end-expiratory or end-inspiratory tail, respectively. This case report outlines a 48-year-old woman with prior subglottic stenosis who presented with recurrent dyspnea. On spirometry, she had the characteristic finding of a flattened end-expiratory tail and was confirmed on imaging to have a left-sided unilateral mainstem bronchial obstruction. Her symptoms improved following a bronchoscopic intervention, and her spirometry pattern returned to normal. Though there are numerous known causes of unilateral mainstem obstruction, the workup for this patient was unrevealing, raising the possibility of idiopathic causes of this disease process. This is a unique case of idiopathic subglottic stenosis and left-sided unilateral mainstem bronchial obstruction occurring in the same patient.

## Introduction

Unilateral mainstem bronchial obstruction is an uncommon cause of dyspnea that can be difficult to diagnose on a clinical exam, especially in the context of existing respiratory pathology. In addition, the tendency of patients to present with respiratory symptoms such as dyspnea, wheezing, and cough may point the clinician in the direction of more common pathologies such as asthma or chronic obstructive pulmonary disease. However, there have been many case reports describing the characteristic biphasic spirometry that can be seen with unilateral mainstem bronchial obstruction from numerous different etiologies.

## Case presentation

**Figure 1:**
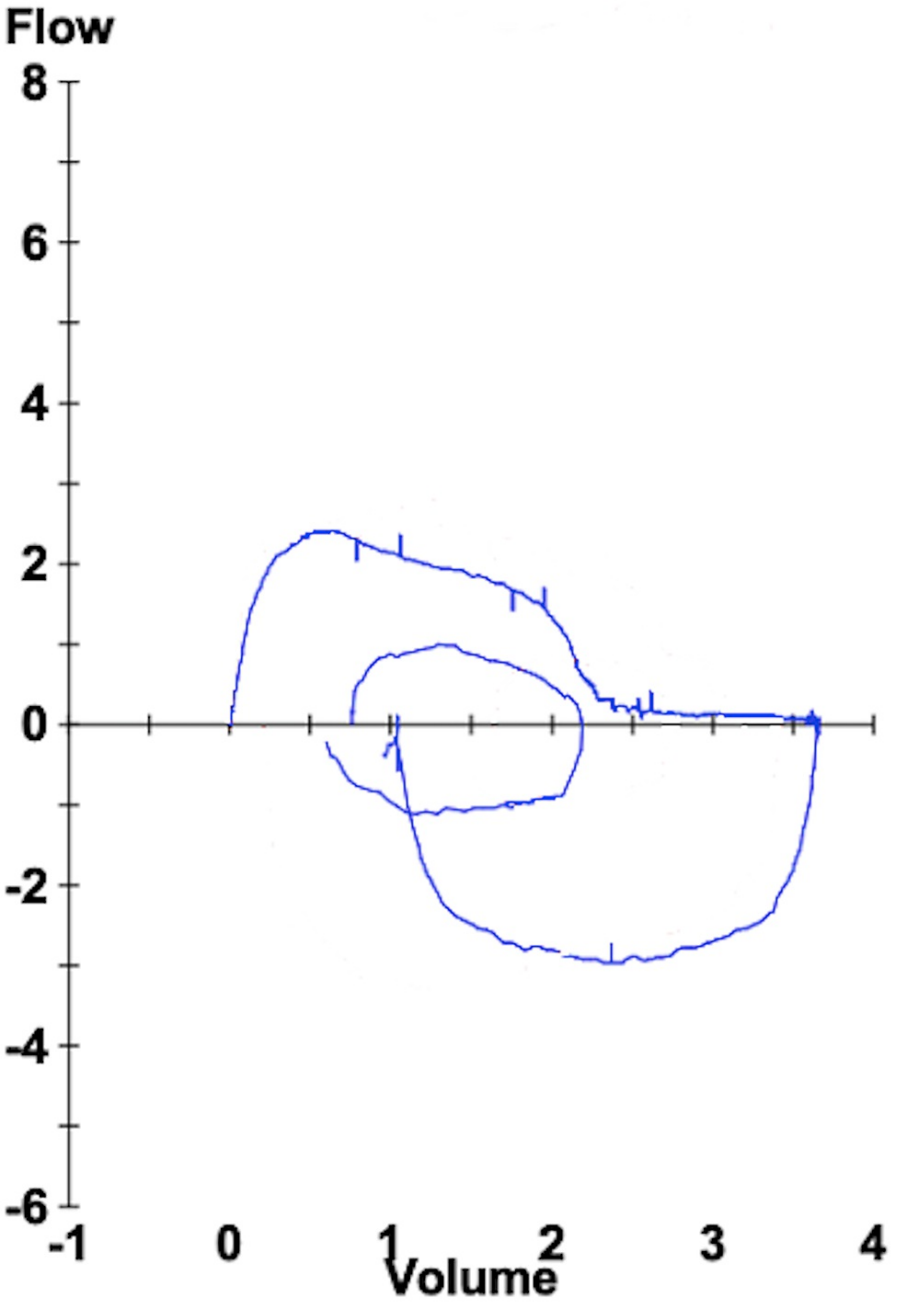
Initial flow-volume loop. Patient flow-volume loop at the time of presentation, showing a decreased expiratory flow followed by a rapid decrease in end-expiratory flow (flattened tail).

The case involves a 48-year-old woman with a history of simple idiopathic grade II subglottic stenosis. Initial symptoms necessitated laryngoscopic balloon dilation at 28 months, 16 months, and six months prior to presentation to the Pulmonary Clinic. Unfortunately, she presented with recurrent dyspnea during minimal exertion. Her flow-volume loop is shown in Figure 1.

The presence of decreased expiratory flow, followed by a rapid decrease in the flow at end-expiration, alerted the treating physicians to the possibility of central airway obstruction in a different location. Her prior history suggested a central (tracheal) obstruction, due to her history of subglottic stenosis.

**Figure 2:**
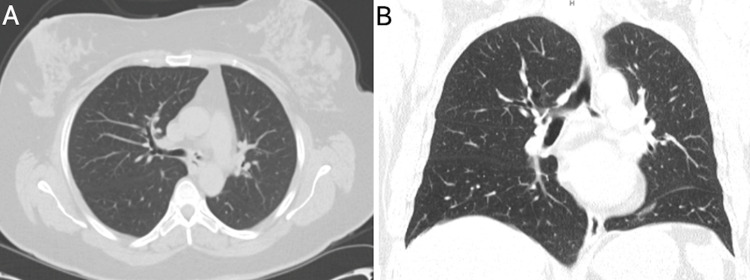
CT of the thorax. (A) Axial cross-section and (B) coronal cross-section of CT of the thorax, showing a narrowing at the level of the proximal left mainstem.

A CT of the thorax (Figure 2) revealed no significant recurrence of subglottic stenosis but new development of severe narrowing of the proximal left mainstem bronchus. The lung parenchyma otherwise appeared normal.

**Figure 3:**
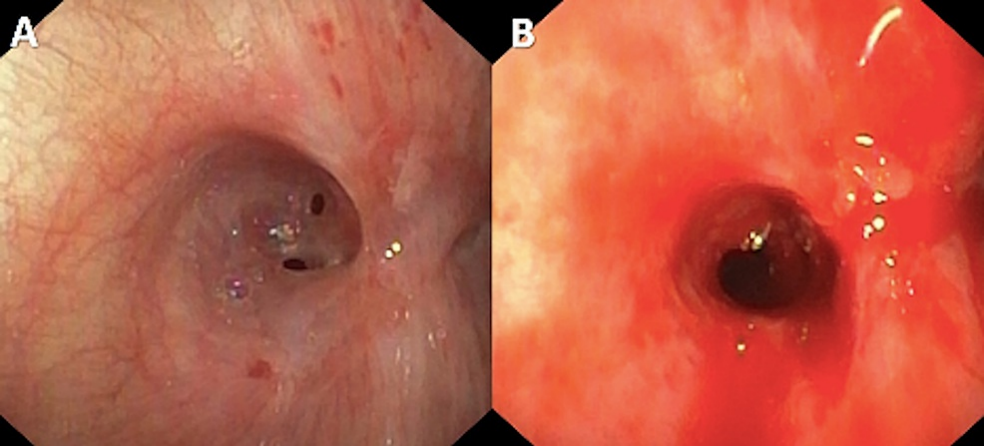
Pre- and post-bronchoscopic intervention. Bronchoscopic view of the left mainstem showing a fibrous, near-complete stenosis (A) before and (B) after bronchoscopic restoration of patency.

The patient underwent flexible bronchoscopy, which confirmed patent right-sided airways but near-complete obstruction of the proximal left mainstem bronchus with complex web-like stenosis. There were two small, 1- to 2-millimeter openings (Figure 3A). Radial incisions were created with an electrocautery knife, followed by serial balloon dilation of the stenotic region up to 10 millimeters (Figure 3B). The distal left mainstem bronchus and lobar bronchi were otherwise patent and normal in appearance.

**Figure 4:**
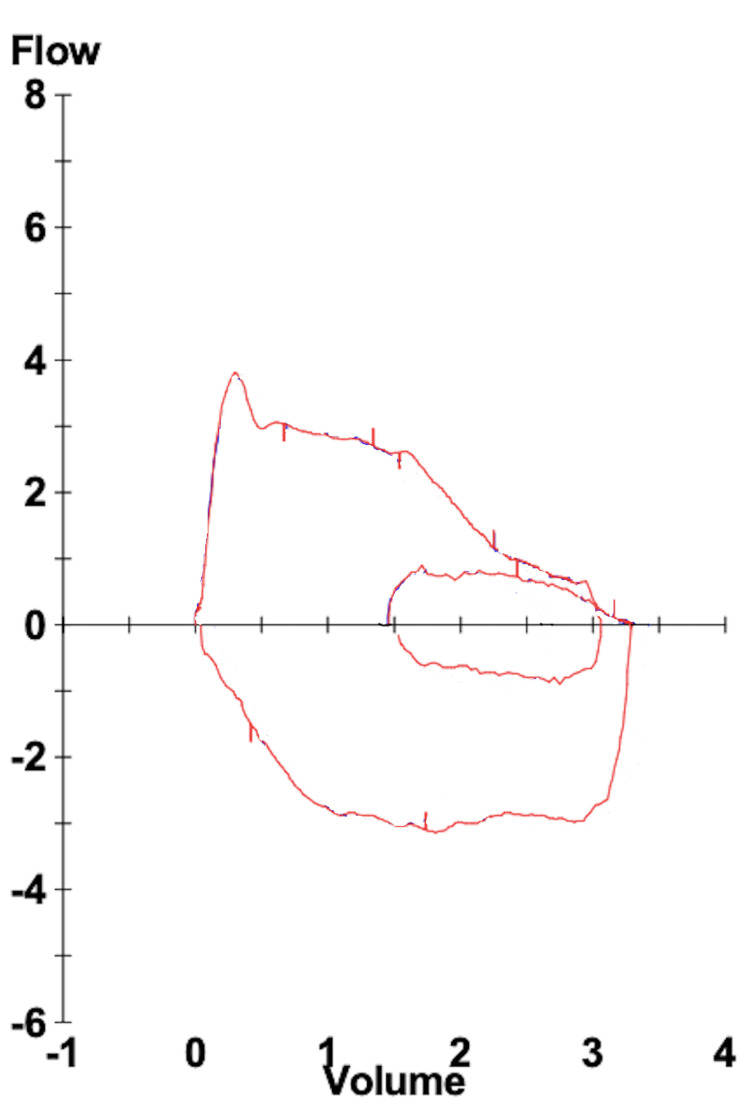
Post-bronchoscopic intervention flow-volume loop. Patient flow-volume obtained three months post-bronchoscopic dilation of the left mainstem obstruction, showing improvement in the expiratory flow and resolution of the end-expiratory tail.

The patient had immediate improvement of her symptoms following the bronchoscopic intervention and returned for spirometry three months later, which showed resolution of the decreased expiratory flow and flattened end-expiratory tail (Figure 4).

## Discussion

Unilateral mainstem stenosis or obstruction can be difficult to recognize as a cause of subacute or chronic dyspnea. However, it has characteristic spirometry findings which can raise suspicion for its presence. These findings were first described by Gascoigne AD et al. in 1990 [1]. Luminal obstruction of a mainstem bronchus has been hypothesized to result in a “two-compartment phenomenon.” The unaffected lung is aerated by the unaffected bronchus and participates in the first part of inspiration and expiration (fast compartment). The affected lung is aerated by the obstructed bronchus (slow compartment) and contributes largely to the later portion of these maneuvers. This is seen as a biphasic expiratory flow-volume loop [2]. There is a low maximal forced expiratory flow, followed by a rapid decrease in flow, which is represented as a flattened end-expiratory tail, as seen in this patient.

Similarly, a normal-appearing inspiratory flow loop may be followed by a flattened end-inspiratory tail. This finding is not as evident in this patient’s spirometry, but has been described in another patient with unilateral mainstem obstruction due to the presence of a bronchogenic cyst [3]. The presence of either or both of these findings is suggestive of unilateral mainstem obstruction and may lead to the diagnoses of disease processes including post-lung transplant stricture, post-infectious stricture, bronchogenic carcinoma, endobronchial metastatic disease, bronchogenic cyst, unilateral emphysema (Macleod syndrome), or developmental cartilaginous ridge, among others [2,3,4].

## Conclusions

Spirometry is an essential tool in determining the etiology of a patient's dyspnea. Though it is most commonly used to diagnose obstructive and restrictive lung diseases, it can also be used to identify the presence of central airway obstruction. Despite this patient's history of tracheal stenosis, she did not have a flow-volume loop with characteristic findings of tracheal flow limitation, such as variable extrathoracic obstruction or fixed obstruction. In this case, the presence of a low maximal forced expiratory flow, followed by a flattened tail at the end of the expiratory flow-volume loop (the two-compartment phenomenon), led to the discovery of a stenotic left mainstem bronchus. In our patient, endobronchial biopsy pathology only showed benign bronchial mucosa and evidence of chronic inflammation. Infectious, rheumatologic, and malignancy workup was unrevealing. To our knowledge, this is the first case of idiopathic subglottic stenosis and idiopathic left-sided mainstem bronchus obstruction occurring in the same patient described in the literature.

## References

[1] Gascoigne AD, Corris PA, Dark JH, Gibson GJ (1990). The biphasic spirogram: a clue to unilateral narrowing of a mainstem bronchus. Thorax.

[2] Agrawal A, Sahni S, Marder G, Shah R, Talwar A (2016). Biphasic flow-volume loop in granulomatosis with polyangiitis related unilateral bronchus obstruction. Respir Investig.

[3] Mazzei JA, Barro A, Mazzei ME, Portas T, Esteva H (2011). Biphasic flow volume curve due to obstruction of main bronchus by bronchogenic cyst. Respir Med.

[4] Teh A, Tan C, Lee CP, Pau J, Seet C, Yap WS (2013). The biphasic flow volume loop - a case report of post-tuberculosis left main bronchus (LMB) stricture. Eur Respir J.

